# An evaluation of the teen and Youth Mental Health First Aid training with a CALD focus: an uncontrolled pilot study with adolescents and adults in Australia

**DOI:** 10.1186/s13033-019-0329-0

**Published:** 2019-11-30

**Authors:** Maria Gabriela Uribe Guajardo, Claire Kelly, Kathy Bond, Russell Thomson, Shameran Slewa-Younan

**Affiliations:** 1 Mental Health, Translational Health Research Institute, Macarthur Clinical School, School of Medicine, Western Sydney University, Locked Bag 1797, Penrith, Sydney, NSW 2751 Australia; 2 Mental Health First Aid Australia, Melbourne, Australia; 3 Centre for Research in Mathematics, School of Computing, Engineering and Mathematics, Western Sydney University, Sydney, Australia; 4 Centre for Mental Health, Melbourne School of Population and Global Health, University of Melbourne, Melbourne, Australia

**Keywords:** Teen and Youth Mental Health First Aid, Culturally and linguistically diverse youth, Mental health, Mental health literacy

## Abstract

**Background:**

Australia is an ethnically diverse nation with one of the largest refugee resettlement programs worldwide. Evidence suggests that although the risk of developing mental disorders in culturally linguistically diverse (CALD) adolescents may be elevated, professional help-seeking in CALD youth is low. This study sought to evaluate the face-to-face teen (tMHFA) and Youth Mental Health First Aid (YMHFA) training with a CALD focus, which aimed at improving mental health literacy (MHL) and skills in youth and adults assisting adolescents with mental health problems.

**Methods:**

An uncontrolled pre-, post-, and follow-up design was used to measure improvement in MHL measures in year 10 students and adults.

**Results:**

A total of 372 year 10 students from 2 high schools were trained. 308 responded to the pre-training questionnaire, 220 responded to the post-training questionnaire, and 256 completed the 3-month follow-up questionnaire. A total of 34 adults were trained, 32 responded to the pre-questionnaire and 31 responded to the post-training questionnaire and 20 completed the 3-month follow-up questionnaire. Following training, students were more likely to endorse ‘helpful’ adults as valid sources of help (p < 0.001) and these gains were maintained at follow-up (p < 0.01). Significantly higher levels of concordant (helpful) helping intentions were found after training (p < 0.01), and this was maintained at follow-up (p < 0.05). Significant lower levels of discordant (harmful) helping intentions were found after training (p < 0.001), and this was maintained at follow-up (p < 0.01). A significant improvement in adults’ knowledge of youth mental health problems and Youth Mental Health First Aid was noted from pre- to post-training (p < 0.01) and was maintained at follow-up (p < 0.01). Confidence when helping a young person with mental health problems increased significantly after training (p < 0.001) and this was maintained at follow-up (p < 0.05).

**Conclusion:**

Our findings indicated the training led to an improvement in a number of measures of MHL and helpful intentions of both the adolescents and adults evaluated. These results indicate that CALD tMHFA and YMHFA are a recommended way of upskilling those trained and thereby leading to the improvement youth mental health in areas with high proportion of ethnically diverse groups.

## Background

### Diversity in Australia

Australia is an ethnically diverse nation with the most recent national census undertaken in 2016 [1] indicating that approximately half (49 per cent) of Australians report having been born overseas (first generation Australian) or had one or both parents that had been born overseas (second generation Australian) [1]. Further, of the 6,163,667 people born overseas, nearly one in five (18%) had arrived since the start of 2012 [1]. Additionally, Australia has one of the largest resettlement programs worldwide [2] providing durable solutions and protection to individuals thought its Humanitarian Visa Program and Permanent Migration Program. It is reported that for the period 2018–2019, the Australian Government will allocate 18,750 places to refugees and others who are displaced as a result of conflict, persecution and human rights abuses [3]. Along with the Humanitarian program, Australia will offer in 2018–2019, a total of 190,000 places which covers skilled, family and special circumstances permanent migration to Australia [3]. Data from the Australian Bureau of Statistics (ABS) indicates that a majority of these ethnically diverse groups choose to resettle in major cities in Australia, and predominately in New South Wales (NSW) (33%) [4]. Relatively, metropolitan Sydney had the largest overseas-born population of all the capital cities [4], concentrated primarily in South Western Sydney, one of the most culturally diverse districts nationwide [5].

### The mental health of culturally and linguistically diverse communities

High prevalence rates of post-traumatic stress disorder (PTSD) and major depression among refugee populations resettled in Western countries have been clearly identified [6]. While reported prevalence rates can vary, data from one of the largest meta-analyses indicated rates of 30.6% and 30.8% for PTSD and depression, respectively [7]. Exposure to high levels of trauma [7] and resettlement challenges (e.g. discrimination, low English proficiency, employment, cultural adjustment) are thought to contribute to poor mental health outcomes in refugee groups [8]. Similarly, migrants often face similar resettlement stressors and as such are at an increased risk of developing mental health conditions [9]. In an Australia-based study [10], it was reported that foreign born (non-English speaking) groups had higher rates of depression (19.7%) compared with Australian counterparts (English speaking), with resettlement challenges being one of the strongest predictor for poor mental health in minorities [9–11]. Equally, migration and resettlement also present challenges for children and youth from migrant, refugee and asylum seeker backgrounds [12].

Exposure to traumatic events and its impact on their mental health—mainly PTSD related problems—have been researched extensively in refugee children and youth [13, 14]. Reported prevalence of PTSD amongst this group varies greatly from 20 to 84% with traumatic exposure demonstrated as being strongest predictor of poor mental health [13]. In addition, literature indicates that migrant children may present with poorer mental health than their peers from the non-migrant population. Stress, anxiety and depression in migrant children are strongly influenced by psychological adaption within the host country [13].

### Mental health literacy in culturally and linguistically diverse communities

It is well-known that adult CALD populations present with complex needs. Further evidence has demonstrated that while these groups are increasingly at risk for psychological distress and mental health problems [8], knowledge about mental disorders, their recognition, management and prevention [15, 16], as well as professional help-seeking remains limited [17]. Evidence suggests that this trend is also present in CALD children and youth [12]. Research has noted that children of CALD backgrounds are often reluctant to seek and report health concerns, due to stigma associated with their minority group status [12].

The term ‘mental health literacy’ (MHL) refers to, ‘knowledge and beliefs about mental disorders which aid their recognition, management or prevention’ [18]. This encompasses (a) the public’s knowledge of how to prevent mental disorders, (b) recognition of when a disorder is developing, (c) knowledge of help-seeking options and treatments available, (d) knowledge of effective self-help strategies for milder problems, and (e) first aid skills to support others affected by mental health problems [18]. Increasing mental health literacy can achieve an important goal of empowering CALD communities and their youth with an understanding of mental disorders, thereby facilitating prevention, early intervention and treatment within their community [19].

### Youth mental health

One marked group at risk of developing mental disorders is young people. The onset of mental disorders usually occurs in childhood or adolescents. Nearly half of all people who experience a mental illness in their lifetime will have had their first episode by the age of 18 [20]. In the general population of Australia, common mental disorders (e.g. anxiety and mood disorders) affect 14% of children and adolescents (aged 8–18 years) over a 12-month period [21]. While the need for early intervention is widely recognised, only a minority of young people with clinically significant symptoms will seek appropriate professional help [22]. It is vital that early and appropriate help is sought because important social, emotional and physical developmental goals occur during adolescence [23]. However, adolescents are known to face several barriers to help-seeking [22], and are also poorly equipped to address the disclosure of a peer’s mental health problem [24]. Thus increasing help‐seeking for adolescents with mental illness is particularly important. Improved help‐seeking in youth and adolescent populations can result in increased likelihood that developmental goals will be attained, may arrest the progression of illness, and can increase the quality of life in those with established mental illness even where pathology remains unaffected by treatment interventions [25].

However, for this to occur, it is essential that adolescents showing symptoms of mental illness are supported to engage appropriate help‐seeking and access effective treatment interventions early in the course of illness. Research on understanding help-seeking in young people identify three possible factors that may play a role: mental health literacy, stigma and social support [26].

### Mental Health First Aid

Mental Health First Aid is ‘the help offered to a person developing a mental health problem, experiencing a worsening of an existing mental health problem or in a mental health crisis. The first aid is given until appropriate professional help is received or until the crisis resolves’ (p. 12) [27]. One established and effective program for increasing mental health literacy, reducing stigma and improving supportive first aid behaviours, is the Mental Health First Aid (MHFA) training provided by Mental Health First Aid Australia [27]. MHFA courses teach about a range of mental disorders such as anxiety disorders (e.g. generalised anxiety disorder), mood disorders (e.g. depression), and crises (e.g. non-suicidal self-injury, panic attacks). This is an evidence-based program that has been found effective in multiple settings and population groups [28]. Relevant to this study are two tailored courses developed by MHFA Australia [29]—the teen MHFA course (aimed at increasing MHL in youth) and Youth MHFA (aimed at increasing MHL in adults who support young people).

### Teen and Youth Mental Health First Aid training

**Fig. 1:**
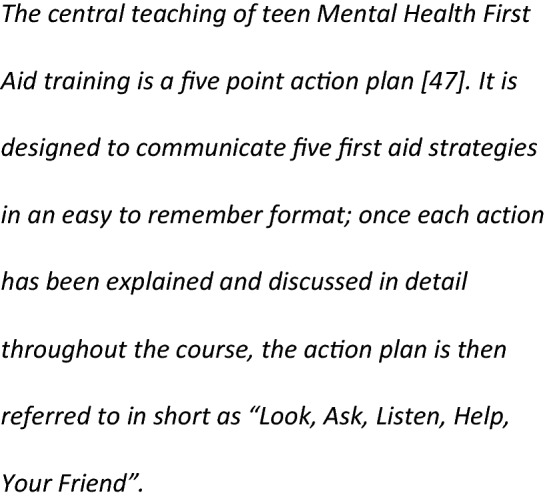
Teen Mental Health First Aid action plan (central teaching)

**Fig. 2:**
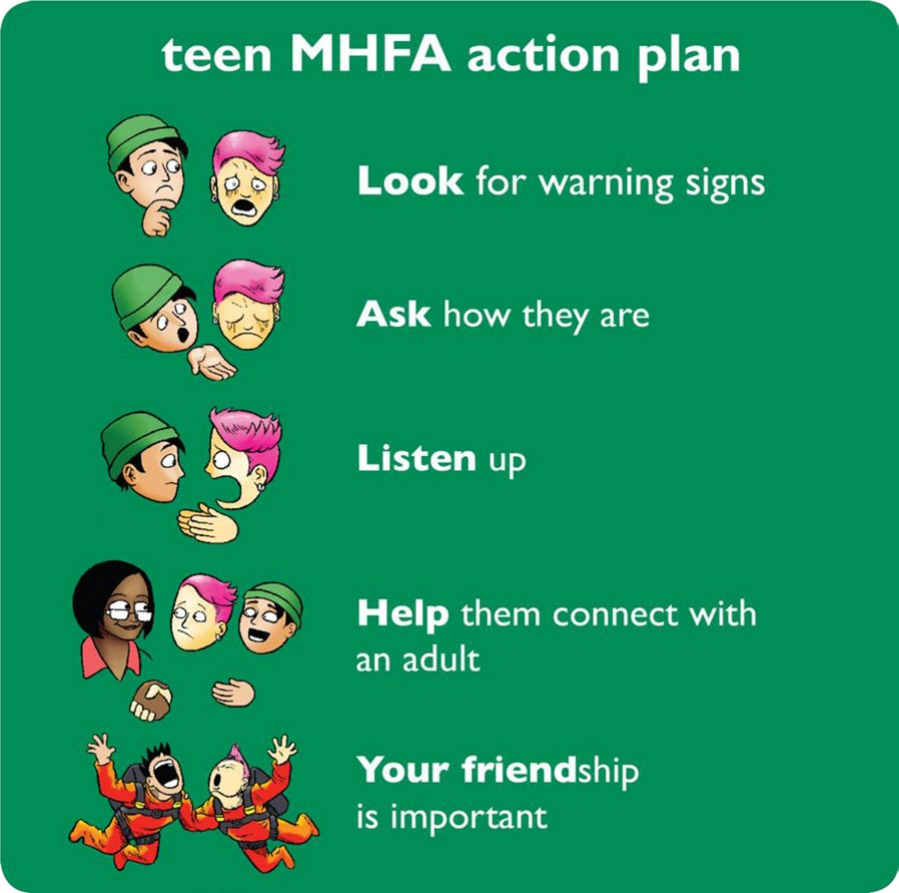
Teen Mental Health First Aid action plan (mnemonic)

The teen MHFA course involves the delivery of a short course to secondary school adolescents in years 10–12. It uses age-appropriate materials developed from research with experts and consumers in the field of youth mental health [29], and consultation with the education sector. The aims of the teen MHFA program is to give young people the skills they need to offer help to a friend experiencing mental health problems or who is in crisis (see Figs. 1 and 2). Broadly, the program focuses on developing knowledge and skills in (a) recognising warning signs that a peer is developing a mental health problem, (b) understanding how to talk to a peer about mental health and seeking help, (c) when and how to tell a responsible adult, (d) where to find appropriate and helpful resources about mental illness and professional help, and (e) how to respond in a crisis situation.

Because a central teaching of the teen MHFA training is to seek assistance from a trusted and reliable adult when a peer is experiencing a mental health problem, the Youth MHFA course is also provided to staff and parents at the students’ schools. This is to ensure that adults who are called upon to assist adolescents are confident in providing support and can facilitate appropriate referral pathways to effective treatment interventions.

### Mental Health First Aid and culturally and linguistically diverse populations

In the last decade, initiatives aiming to improve MHL in adult refugee and migrant populations living in Western countries [30–32] have been developed. In Australia, adapted versions of the MHFA training has been delivered to improve mental health literacy in members of the Chinese [30] and Vietnamese [31] community in Australia. Equally important, this successful training model has been adapted also to improve MHL of staff working with refugee communities in Australia [32].

Overseas this training has also been used in community-based interventions in the US [33, 34]. Subedi et al. evaluated an MHFA course delivered to Bhutanese refugees resettled in the US, reporting positive changes around knowledge of appropriate help-seeking and useful interventions (more concordant with professionals’ beliefs) for mental health problems.

More recently, an 8-h adapted version of the MHFA training has been delivered and evaluated in community-based workers assisting underserved Latin and Asian American immigrant communities in the US [34]. Findings revealed that the MHFA training was successful at helping advocates to recognize signs and symptoms of mental disorders and increasing confidence when helping immigrants who may develop mental health problems [34].

While community-based level initiatives to improve MHL in CALD populations are growing, providing training on MHFA skills to CALD adolescents (or their first contact supports) are rare, with much of the current work focused on clinical-based interventions [35]. There is clearly a need for an early intervention approach seeking to increase MHL of CALD youth at a community-level.

The aim of the current study was to conduct an uncontrolled pilot evaluation of the teen and Youth MHFA with a CALD focus on measures of knowledge, attitudes and behaviours towards mental health crises or problems in year 10 students and responsible adults.

## Methods

### Participants

The training was delivered in the Local Government Area of Fairfield, located in South Western Sydney, where a high proportion of adolescents with CALD background are enrolled in local high schools. Participants of this evaluation study were year 10 students and responsible adults (parents and teachers) from two high schools located in Fairfield. These schools were selected on the basis of their location (within Fairfield area), the access to a high number of students with CALD background and their capacity to host the research throughout 2018 (e.g. able to provide computers for their students to complete surveys on, have classrooms equipped with audio-visual equipment, and have a timetable which would accommodate the teen MHFA program).

#### Students

Year 10 was selected as the target group because these students are considered to be located between the junior years (years 7–9) and senior years (years 11–12) and may have contact with both groups. All year 10 students attending the teen MHFA training were invited to participate in the evaluation surveys by their year coordinators or the student wellbeing staff. Passive parental consent (“opt-out consent”) with adolescent assent was obtained for this research.

A power analysis on the number of year 10 students required was undertaken to inform the sample size required for the evaluation. Using Cohen’s effect size estimates for statistical analyses in the behavioural sciences and making the conservative assumption of no correlation between pre- and post- tests, a sample of 198 participants was calculated to give 80% power to detect a small effect size (d = 0.2) from pre to post-test with alpha = 0.05 (Sample Power 3.0). This power analysis was a considered balance between reducing probability of Type I and Type II error, allowing enough power to detect plausible effects, and selection of a suitable, achievable sample size.

A total of 372 year 10 students were trained. Eighty-three percent of total number trained responded the pre-training questionnaire, 59% responded to the post-training questionnaire, and 69% completed the 3-month follow-up questionnaire.

#### Teachers/responsible adults

All teachers/responsible adults attending the YMHFA training were invited to participate in the research evaluation component. A power analysis is not calculated for the YMHFA training as we recognise the numbers may be underpowered, however the primary purpose of this training is to support the year 10 students.

A total of 34 adults were trained, 94% responded the pre-questionnaire and 91% responded the post-training questionnaire and, 59% completed 3-month follow-up questionnaire.

Approval for this research was granted by the Western Sydney University Human Research Ethics Committee (reference number H12695) and the Department of Education (SERAP number 2018334).

### Interventions

#### Teen Mental Health First Aid

The teen MHFA training intervention involves three 75-min classroom sessions facilitated by an accredited teen MHFA Instructor with specific training and experience in youth mental health. Sessions are presented to regular class groups of between 15 and 30 students. Training is normally completed within 5 to 8 school days, depending on timetabling at each school, with at least 1 day between each session. The training involves: a PowerPoint presentation, videos, role plays, group discussion and small group activities. A student booklet is provided for each participant, for use in sessions and for reference following the completion of the course [36]. All instructor training is supplemented with a teaching manual in order to guide facilitation and ensure fidelity and consistency.

Table 1 outlines the teen Mental Health First Aid course content.

**Table 1:** Structure and content of the teen Mental Health First Aid training

Session 1: 75 min	Session 2: 75 min	Session 3: 75 min
Mental health problems Topics presented: • What is mental health? • What are mental health problems? • Types of mental health problems • Impact on young people • Stigma • Appropriate help	Helping a friend in a mental health crisis Topics presented: • What is mental health first aid? • What is a mental health crisis? • Using the teen MHFA action plan to help a friend in crisis • Recovery position	Helping a friend who is developing a mental health problem Topics presented: • Helping a friend who is developing a mental health problem • Importance of acting early • Using the teen MHFA action plan to help a friend developing a mental health problem • Helpful links and resources
Videos: • Talking about it 1 (4:50 s) • Getting help (5:32)	Video: • Mates (13:55)	Videos: • Talking about it 2 (4:14) • Talking about it 3 (6:02)
Activities: • Group discussion of how mental health problems impact on young people • Identifying supportive adults • Relaxation	Activities: • Group discussion of confidentiality vs safety • Role play recovery position	Activities: • Group discussion of Luke and Ali’s stories • Role play using the action plan

#### Youth Mental Health First Aid

The 14-h YMHFA course, launched in 2007, teaches adults how to support adolescents who might be developing a mental health problem or in a mental health crisis, and to assist them to receive professional help. The course content and manual are modified to provide information which is specific to adolescents [37]. In addition to the mental health problems covered in the Standard MHFA course [27], YMHFA covers eating disorders and non-suicidal self-injury.

There is a strong theme throughout the whole program about the importance of early intervention to minimise the impact of mental health problems on adolescent development. The course can be delivered flexibly as either 2 full days (which do not have to be consecutive) or over four sessions of 3.5 h each. Targets for the training include parents, school professionals, adults involved in recreational activities with adolescents (e.g., sport coaches and scout leaders) and other adults who work with or care about adolescents. The Action Plan does not differ from that provided in the Standard MHFA course [Ref standard MHFA manual], although the application is tailored to the needs of adolescents.

#### Adaptation to resources incorporated to the curriculum

The Youth and teen MHFA courses for CALD community included the development of new case scenarios which better represented the needs of CALD young people, the development of a resource list with relevant local services, and two videos featuring interviews with mental health professionals in their local area. The first of the two videos was designed to help adults to tailor their communication to the needs of young people from a different cultural background to their own, and the second assisted adults to adjust their communication to the needs of students who have (or are like to have) a history of trauma as part of their migration experience, e.g. those who have arrived as refugees and have experienced unrest in their country of origin and those who have experienced war or torture. The courses retained all of the elements of the non-CALD Youth and teen MHFA programs and the Action Plans were not altered in any way. An advisory group was convened to contribute their expertise and feedback on all culturally adapted resources. In addition, by ensuring that the instructors of both the Youth and teen MHFA programs were from CALD backgrounds, we were able to ensure that language and examples were tailored to the audience.

### Measures

The surveys were distributed online hosted by www.qualtrics.com. The questions were developed to measure mental health literacy, stigmatising attitudes, MHFA behaviours, and the help-seeking status of adolescents in year 10. Within the adult group, questions were designed to measure mental health literacy, stigmatising attitudes, MHFA behaviours, and appropriate help-seeking and youth mental health knowledge.

Surveys were administered at three time points: before, immediately after, and 3 months following completion of the training (see Table 2).

**Table 2:** Variables measured in year 10 students and teachers/responsible adults across time

Variable measured	Pre-training	Post-training	3-Month
Demographics	✓		
Mental health literacy			
Recognition of mental health problem—general	✓	✓	✓
Adults thought to be helpful	✓	✓	✓
Knowledge of youth mental health quiz^b^	✓	✓	✓
Stigmatising attitudes			
Social distance	✓	✓	✓
Stigma			
Weak-not-sick subscale	✓	✓	✓
I would not tell anybody	✓	✓	✓
Dangerous/unpredictable subscale	✓	✓	✓
MHFA intentions and behaviours			
Confidence helping	✓	✓	✓
Offering help^b^	✓	✓	✓
MHFA intentions—helpful^a^	✓	✓	✓
MHFA intentions—harmful^a^	✓	✓	✓
MHFA intentions—ALGEE score^b^	✓	✓	✓
MHFA experiences—provided to peer^a^	✓		✓
MHFA experiences—received from a peer^a^	✓		✓
MHFA experiences—ALGEE score^b^	✓		✓

^a^Only measured in year 10 students

^b^Only measured in teachers/responsible adults

The questionnaires included items adapted from the Australian National Survey of Youth Mental Health Literacy [38], and were focused on a hypothetical case vignette of a CALD adolescent experiencing social anxiety symptoms (Eman). The vignette is provided in appendix 2. All the open-ended responses were coded by a researcher (GU).

### Mental health literacy

#### Recognition of mental health problems

Problem recognition was assessed by asking participants to identify what, if anything was wrong with Eman (the character in the vignette). Responses were open-ended. The labels given to these vignettes have been previously validated against the diagnoses of mental health professionals [39]. Coding for recognition of mental health problem was based on responses using key words. The labels were included in the ‘*mental health problem*’ category were ‘*anxiety*’, ‘*anxious*’, ‘*depression*’, ‘*mental illness*’, ‘*mental disorder*’, ‘*mental problem*’, ‘*trauma*’ and/or *‘traumatic*’.

#### Adults thought to be helpful

Both groups of participants were asked to rate a range of potential sources of help as likely to be helpful for ‘Eman’. Potential sources of help included: close friend, counsellor, family member, general practitioner, minister/priest, parent, psychologist, school counsellor and teacher. These items were used to measure belief in seeking adult help, which is a key message of the training [36, 37]. Scores range from 0 to 6, with 1 point assigned for endorsing each helping adult.

#### Knowledge of youth mental health problems in teachers/responsible adults

Knowledge of mental health problems was measured by an 18-item questionnaire specifically designed to cover information in the course. This was a modified version of a questionnaire previously used in MHFA evaluation trials [40]. The questionnaire included statements reflecting general knowledge of youth mental health. Some examples of these items were ‘*Recovery from anxiety disorders requires teenagers to face situations which are anxiety provoking*’; ‘*Antidepressant medications can be an effective treatment for most anxiety disorders*’ *and* ‘*Cognitive behaviour therapy (CBT) can help relieve depression in teenagers*’. Response options for each item were ‘Agree’, ‘Disagree’ or ‘Don’t know’. Scoring was based on 1 point per correct response, providing a maximum score of 18.

### Stigmatising attitudes

#### Personal stigma

Both participant groups were asked to respond to seven questions assessing personal stigma towards ‘Eman’. These questions were measured using a Likert scale (1 = ‘strongly disagree’ to 5 = ‘strongly agree’). The questions were: (1) Eman could snap out of it if (she) wanted; (2) Eman’s problem is a sign of personal weakness; (3) Eman’s problem is not a real medical illness; (4) Eman is dangerous to others; (5) It is best to avoid Eman so that you don’t develop this problem yourself; (6) Eman’s problem makes (her) unpredictable; (7) If I had a problem like Eman’s I would not tell anyone.

#### Social distance

An additional five items were adapted for the student group from the Social Distance Scale [41, 42]. These questions asked whether the participant would be happy to: (1) develop a close friendship with Eman; (2) go out with Eman on the weekend; (3) go to Eman’s house; (4) invite Eman around to their house; (5) work on a project with Eman. Each question was rated on a 4-point Likert scale (1 = ‘yes definitely’ to 4 = ‘definitely not’). Higher scores in both personal stigma and social distance measures denoted higher negative attitudes towards mental health problems.

### Mental Health First Aid intentions and behaviours

#### Confidence when helping someone with a mental health problem

Confidence with providing mental health first aid, considered the primary outcome of interest, was assessed by asking how confident (using a 5-point Likert scale) both the student and adult participants felt in helping the person in the vignette. Scores ranged from 1 to 5 with higher scores reflecting greater degree of confidence.

#### Offering help

To measure willingness to offer help responsible adults/teachers were asked ‘*Eman was one of your students, I would help her*’. Rating for this item was made on a 7-point Likert scale (‘1 = strongly disagree’, ‘2 = mostly disagree’, ‘3 = somewhat disagree’, ‘4 = neither agree nor disagree’, ‘5 = somewhat agree’,’ 6 = mostly agree’, ‘7 = strongly agree’).

#### Mental Health First Aid intentions

Mental Health First Aid intentions were assessed in year 10 students by asking: ‘*If Eman was someone you knew and cared about, what would you do to help (her)?*’. There was a total of 12 possibilities, 6 were consistent (rated as helpful) with the action plan taught in the course and 6 were discordant (harmful) with the action plan. The total score ranged from 0 to 6 in each subscale. Higher scores in the helpful subscale mean higher quality of helping intentions whereas, in the harmful subscale higher scores reflected poorer quality of helping intentions.

In the adult group, participants were asked to ‘Describe all the things you would do to help Eman’. De-identified responses were scored by a researcher (GU). A quality scoring system was utilised to measure the quality of these helping intentions devised by Yap and Jorm [43].

This scoring system is based on the MHFA Action Plan taught in the fourth edition of the MHFA course [27]. Responses are awarded a point for each component of the Action Plan they mention (i.e. Approach the person, Assess and Assist with any crisis, Listen and communicate non-judgementally, Give support and information, Encourage appropriate professional help and Encourage other supports: ALGEE score) and an additional point per category where specific details are given (e.g. ‘Encourage the person to see a psychologist’ would receive two points for Encourage appropriate professional help). Responses ranged from a minimum of 0 to a maximum of 2 points per component. This resulted in total score representing quality of help intention that ranged from 0 to 12. This score has previously been found to predict quality of subsequent helping behaviours, indicating its validity [44].

#### Mental health first aid experiences

Adolescents’ mental health first aid experiences were assessed at pre-training and follow-up by asking if in the last 3 months they had contact with anyone who they thought might have a mental health problem or experienced a mental health crisis. A mental health problem was defined as a major change in a person’s normal way of thinking, feeling or behaving, which interferes with the person’s ability to get on with life, and does not go away quickly or lasts longer than normal emotions or reactions would be expected to. Participants were told that this might involve a diagnosed mental illness, a worsening of a mental health problem, an undiagnosed problem, or a drug or alcohol problem [29]. A mental health crisis was defined as when a person is at increased risk of harm to themselves or to others. Participants were told that crisis situations might include having thoughts of suicide, engaging in self-injury, being very intoxicated with alcohol or other drugs, or experiencing bullying or abuse.

Participants who indicated having contact with a peer with a mental health problem were asked ‘What did you do to help the person?’. For this question, a series of consistent helping behaviours based on the action plan were presented. In addition, students were asked about their own mental health and if they had received help in the past. For those who responded ‘yes’ to both of these questions, a third question ‘Who provided support or help for the problem?’ was displayed. Further, if they were helped by a ‘peer’ (friend), they were next asked to select multiple options of ‘What did your friend do to help you?’. Again, these options were consistent with helping behaviours based on the action plan taught in the course. Scales of help provided to or received from a peer ranged from 0 to 6 points, where higher scores indicated higher quality of help provided or received.

In order to assess MHFA experiences in the adult group, teachers/responsible adults (only) were asked ‘over the last 12 months, has any young person (12–18) you know had any sort of mental health problem?’ at pre-training and 3-month follow-up. Participants were also asked to describe all the things they did to help the person (adolescent) retrospectively at pre-training and 3-month follow-up. Open-ended response rating was performed by a research assistant (GU) based on the scoring system (ALGEE score) devised in a previous study [40].

### Statistical analysis

A mixed effects model was used to assess the differences between pre- and post-, and pre- and follow-up measures. For the binary outcome measures, a logistic mixed effects model was used, and the effect sizes were presented as odds ratios. For the continuous outcome measures, a linear mixed effects model was used, and the effect sizes were presented as marginal differences in the means (or the beta coefficient for the effect of age). p-Values were calculated from the Wald’s tests. Analyses were performed using R (v3.5.1 Feather Spray) using the functions lme and glmmPQL in libraries nlme and MASS respectively. Multiple imputation was used to account for all missing data, using predictive mean matching for continuous variables, and logistic regression for categorical variables. The number of imputations performed per analysis was 20, and the results were pooled using Rubin’s method [45]. The R library, mice was used to perform the multiple imputation.

Finally, paired sample t tests were used to assess changes on actual helping behaviours, which were administered at two time points, pre-training and 3 months after the training completion for both participant groups. For the adolescent group MHFA experiences provided to or received from a peer was compared at pre-training to that reported at 3-month follow-up. For the adult group, MHFA experiences provided to an adolescent by a teacher/responsible adult was compared pre and follow-up. This analysis was undertaken using SPSS version 25. An alpha level of 0.05 for all statistical tests was used.

## Results

**Fig. 3:**
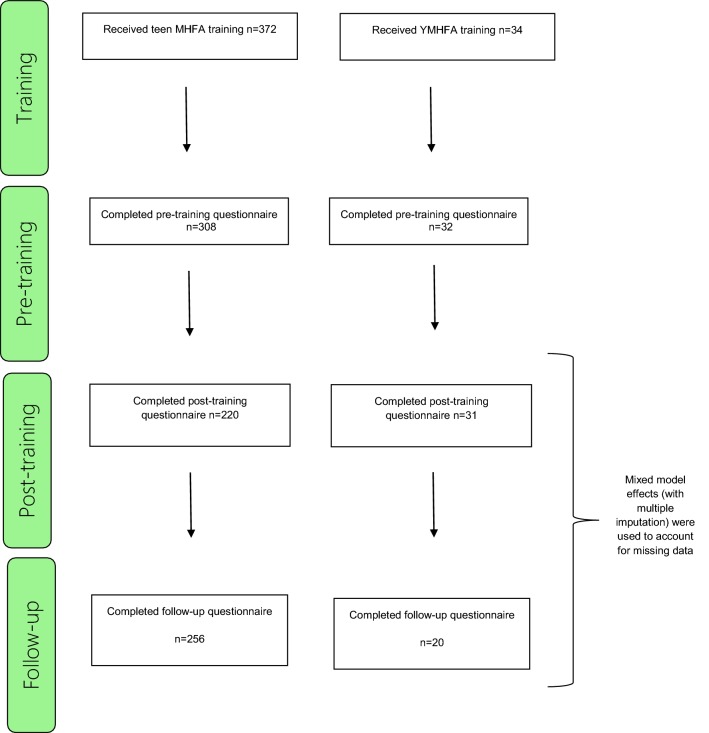
Teen and youth MHFA participants’ flowchart

Figure 3 presents the training and evaluation numbers for the year 10 students and responsible adults. Demographic characteristics of both groups are presented in Table 3.

**Table 3:** Demographics characteristics of year 10 students and responsible teachers

Characteristics	Pre-training (n = 308)^a^	%
Adolescent group		
Gender		
Male	148	48.2
Female	155	50.5
Identify with another term	4	1.3
English as first language		
Yes	145	47.1
No	163	52.9
Language other than English (top 3)		
Vietnamese	59	21.9
Assyrian	22	7
Arabic	20	6.4
Age (years old)		
14	1	3
15	221	71.8
16	81	26.3
17	5	1.6
14 years old or older in 2018		
Yes	295	95.8
No	13	4.2

	Pre-training (n = 34)^b^	%
Teachers/responsible adults group		
Gender		
Male	9	28
Female	23	72
Age (years old)	38.38 (13.50)	–
Occupation		
Teacher	28	87
Other	4	13
Qualifications		
Certificate	2	6.3
Bachelor degree	19	59.4
Masters degree	11	34.3

^a^May not add to 308 due to missing data

^b^May not add to 34 due to missing data

### Teen Mental Health First Aid

Table 4 presents detailed data on participants’ recognition, knowledge, attitudes towards mental health problems and helping behaviours across pre-training, post-training and 3-month follow-up.

**Table 4:** Students’ data across time

Variables	Pre-training	Post-training	Follow-up	Mean difference for pre- versus post	OR for pre versus post	Mean difference for pre versus follow-up	OR for pre versus follow-up
Mental health literacy							
Problem recognised as ‘mental health problem’^a^ (%)	35.90%	39.70%	36.60%	–	1.81	–	1.03
Adults thought to be helpful (mean, CI 97.5)	3.53 (3.77)	4.26 (4.49)	3.95 (4.19)	0.72***	–	0.41**	–
Stigmatising attitudes							
Social distance scale (mean, CI 97.5)	9.73 (10.27)	9.46 (9.94)	9.19 (9.64)	0.25	–	0.53	–
Personal stigma (mean, CI 97.5)							
Weak-not-sick subscale	2.23 (2.33)	2.12 (2.24)	2.09 (2.20)	0.11	–	0.13*	–
I would not tell anybody	2.57 (2.70)	2.42 (2.56)	2.53 (2.67)	0.14		0.03	
Dangerous/unpredictable subscale	2.21 (2.32)	2.13 (2.24)	2.15 (2.25)	0.08	–	0.05	–
MHFA intentions and behaviours							
Confidence helping (mean, CI 97.5)	3.59 (3.71)	3.68 (3.82)	3.66 (3.80)	0.09	–	0.07	–
MHFA intentions—helpful (mean, CI 97.5)	4.21 (4.43)	4.63 (4.81)	4.48 (4.67)	0.41**	–	0.27*	–
MHFA intentions—harmful (mean, CI 97.5)	1.72 (1.86)	1.37 (1.52)	1.45 (1.58)	0.34***	–	0.25**	–
MHFA experiences—provided to a peer (mean, SD)^b^	3.32 (1.53)	–	3.68 (1.71)	–	–	0.36	–
MHFA experiences—received from a peer (mean, SD)^b^	2.62 (1.71)	–	2.69 (1.65)	–	–	0.07	

^a^Multiple responses are permitted

^b^Completers only, *p < 0.05 **p < 0.01 ***p < 0.001

### Mental health literacy

#### Recognition of mental health problems

To assess whether recognition of the problem in the vignette as just a ‘general mental health problem’ improved over time, the frequencies of all other responses representing a mental health related label (‘anxiety’, ‘anxious’, ‘depression’, ‘mental illness’, ‘mental disorder’, ‘mental problem’, ‘trauma’ and/or ‘traumatic’) were included. 35.9% of students recognised the problem in the vignette as ‘general health problem’, 39.7% of them did after training and 36.6% of them did at follow-up. This small increase was not significant across times.

#### Adults thought to be helpful

Following training, students were more likely to endorse ‘helpful’ adults as valid source of help (*p *< 0.001) and these gains were maintained at follow-up (p < 0.01).

### Stigmatising attitudes

Preferred social distance from a peer with a mental health problem was not significantly reduced following training or at follow-up. However social distance scores were quite low to begin with and there may have been a ceiling effect in place.

Lower levels of stigma in the weak-not-sick subscale were found after training, although this was not significant. However, a significant reduction in the weak-not-sick subscale was found at 3-month follow-up (p < 0.05). ‘*I would not tell anybody*’ subscale and ‘*Dangerous/unpredictable*’ subscale scores were not reduced significantly after training or at follow-up.

### Mental health first aid intentions and behaviours

#### Confidence in providing help

Although confidence when helping a peer with mental health problems slightly increased following training this was not significant at post training or at follow-up.

#### MHFA intentions—helpful

Significant higher levels of consistent (helpful) helping intentions were found after training (p < 0.01), and this was maintained at follow-up (p < 0.05).

#### MHFA intentions—harmful

Significant lower levels of discordant (harmful) helping intentions were found after training (p < 0.001), and this was maintained at follow-up (p < 0.01).

#### MHFA experiences—provided to a peer

A total of 75 students provided responses to indicate that they had in fact actually tried to help a peer and selected the interventions that allowed for measure of helping behaviours to be scored, with a comparison undertaken between pre-training and follow-up time points. Although not statistically significant, there was a slight increase in scores between the two time points.

#### MHFA experiences—received from a peer

A total of 13 students provided responses to indicate that they had in fact actually received help from a peer and selected the received interventions that allowed for measure of helping behaviours to be scored, with a comparison undertaken between pre-training and follow-up time points. Although not statistically significant, there was a slight increase in quality of help received from a peer between the two time points.

#### Youth Mental Health First Aid

Table 5 presents detailed data on participants’ recognition, knowledge, attitudes towards mental health problems and helping behaviours across pre-training, post-training and 3-month follow-up.

**Table 5:** Teachers/responsible adults’ data across time

Variables	Pre-training	Post-training	Follow-up	Mean difference for pre versus post	OR for pre versus post	Mean difference for pre versus follow-up	OR for pre versus follow-up
Mental health literacy							
Problem recognised as ‘mental health problem’^a^ (%)	84%	93%	89%	–	2.32	–	1.50
Adults thought to be helpful (mean, CI 97.5)	4.07 (4.63)	4.81 (5.59)	4.49 (5.21)	0.73*	–	0.41	–
Knowledge of mental health problems (quiz)	9.26 (10.37)	11.67 (12.85)	11.30 (12.57)	2.41**	–	2.03**	–
Stigmatising attitudes							
Social distance scale (mean, CI 97.5)	8.21 (9.12)	7.90 (8.82)	8.95 (10.09)	0.39	–	0.23	–
Personal stigma (mean, CI 97.5)							
Weak-not-sick subscale	1.65 (1.89)	1.58 (1.82)	1.93 (2.24)	0.06	–	0.28	
I would not tell anybody	1.84 (2.13)	1.54 (1.82)	1.53 (2.02)	0.30	–	0.31	–
Dangerous/unpredictable subscale	1.50 (1.67)	1.41 (1.59)	1.35 (1.57)	0.11	–	0.15	–
MHFA intentions and behaviours							
Confidence helping (mean, CI 97.5)	3.92 (4.11)	4.37 (4.56)	4.25 (4.48)	0.44***	–	0.32*	–
Offer help	4.40 (4.71)	4.64 (4.85)	4.47 (4.83)	0.24	–	0.07	–
MHFA intentions—ALGEE score	3.17 (4.02)	4.24 (5,12)	4.08 (5.10)	1.06		0.90	
MHFA experiences^b^—ALGEE score	2.14 (0.37)	–	2.57 (1.13)	–	–	0.42	–

^a^Multiple responses are permitted

^b^Completers only, *p < 0.05 **p < 0.01 ***p < 0.001

### Mental health literacy

#### Recognition of mental health problems

To assess whether recognition of the problem in the vignette as a ‘*general mental health problem*’ improved over time, the frequencies of all other responses representing a mental health related label (‘*anxiety*’, ‘*anxious*’, ‘*depression*’, ‘*mental illness*’, ‘*mental disorder*’, ‘*mental problem*’, ‘*trauma*’ and/or ‘*traumatic*’) were included. Results indicated that 84% of adults recognised the problem in the vignette as ‘*general mental health problem*’, this increased to 93% after training, moving to 89% at follow-up. However, this small increase was not significant across times.

#### Adults thought to be helpful

Following training, teachers/responsible adults were more likely to endorse ‘helpful’ adults as valid source of help (*p *< 0.05). However, these gains were not maintained at follow-up.

#### Knowledge of mental health problems

A significant improvement in participants’ knowledge about youth mental health problems and Youth Mental Health First Aid was noted from pre- to post-training (p < 0.01) and were maintained at follow-up (p < 0.01).

### Stigmatising attitudes

Stigma, as measured by social distance and attitudes (weak-not-sick, ‘I would not tell anybody’, and ‘Dangerous/unpredictable’) were low at baseline and did not improve. This may be due to a ceiling effect.

### Mental health first aid intentions and behaviours

#### Confidence helping

Confidence when helping a young person with mental health problems increased significantly after training (p < 0.001) and this was maintained at follow-up (p < 0.05).

#### Offering help

Willingness to offer help to a young person with mental health problems was not significantly increased following training or at follow-up.

#### MHFA intentions—ALGEE score

Although helping intentions increased from pre-training to after-training and from pre-training to 3-month follow-up, these differences were not significant.

#### MHFA experiences—ALGEE score

A total of 7 teachers/responsible adults provided responses to indicate that they had in fact actually tried to help a young person and described what they did that allowed for a measure of helping behaviours to be scored, with a comparison undertaken between pre-training and follow-up time points. Although not statistically significant, there was a slight increase in scores between the two time points, despite a narrow follow-up of 3 months.

## Discussion

The current study sought to evaluate whether the teen and Youth MHFA with a CALD focus was effective in changing participants’ knowledge, intentions, confidence, attitudes and behaviours. In the student group, our results demonstrated a significant impact on some of the key MHL measures, such as increasing knowledge of helpful adults, improving participants’ intention to help and decreasing some negative attitudes.

Significant improvements in student’s knowledge of who are considered helpful adults when seeking help for mental health concerns was increased in students following training and maintained at follow-up. This improvement is a reflection of the emphasis the teen MHFA has on encouraging to trust and disclose personal issues or mental health concerns to responsible adults that can provide help at different levels (teacher, school counsellor, psychologist) [36]. Our findings are in line with results arising from a previous teen MHFA evaluation study conducted by Hart et al. [29] where training impacted positively and improving report of ‘helpful ‘adult sources (e.g. GP, counsellor) overtime. Encouraging young people to keep an eye out for their friends and offer help when they notice worrying changes is the core message in the training [36]. Trusting informed adults is key for ‘close support’, ‘providing hope’, ‘recognition’, ‘prevention’, ‘treatment’ or ‘management of a mental disorder’. This noted improvement is a positive achievement, especially considering young people are reluctant to trust adults or disclose their feelings for fear of being judged, especially when the conversation involves suicide or non-suicidal self-injury [46, 47]. This is likely to be more pronounced in students with CALD backgrounds, as they are can be relatively new to the country, still forming relationships and getting to know new systems, culture, language and their school teachers, support staff or health providers [12]. Feelings of shame or distrust when disclosing mental health concerns in minority groups have been well-identified [12] adding a new layer to the already complex help-seeking process. Thus this increase represents positive achievement of this training program to assist CALD students.

Another marked contribution of the teen MHFA was the significant increase in the knowledge of students around positive (concordant) helping intentions towards a peer with mental health problems after training and at follow-up. These positive helping intentions (e.g. encourage the friend to talk to a health professional or other adult) are in line with what research has demonstrated to be the best first aid actions and are consistent with the teen MHFA teaching content (and the action plan). Similarly, a significant reduction of negative (discordant) helping intentions as best first aid actions was found after training and was maintained at follow-up. Negative intentions such as ‘try to deal with it on my own’ or ‘do nothing’ are at odds with the early intervention paradigm and create a barrier to mental health service provision. Increasing the knowledge around helpful and harmful interventions is particularly relevant in CALD populations given previous research has demonstrated a preference for ‘dealing with mental health problems on their own’ was selected by large proportion of refugees in an Australian-based study [15].

Unfortunately, not all measures were found to improve following training. One such example was students’ MHFA behaviours which demonstrated non-significant improvement. A number of reasons could account for this. Firstly, students reporting undertaking such behaviours (experiences) were fewer in number, a reflection of being less willing to respond such questions which was also noted in Hart et al. [29]. Additionally, the short follow-up period (3 months) may have been too brief to have allowed more students the opportunity to put into action their newly acquired skills in this regard.

In the teachers/responsible adults receiving the Youth MHFA, the training improved confidence when helping young people and increased knowledge about youth mental health across times.

Knowledge about mental health problems experienced by youth is crucial when trying to assist a young person. The Youth MHFA training was found to impart information about youth mental health and Youth Mental Health First Aid strategies. Participants were better able to identify and endorse evidence-based interventions for depression and anxiety (e.g. CBT, exposure therapy, medication for severe cases). Additionally, it provided guidelines on how to approach and interact with teenagers experiencing a range of mental health problems including psychosis or who are misusing alcohol or other drugs (e.g. cannabis). It also encouraged participants to support adolescents even if they do not want any help. The training also provides accurate information about how to approach a young person with suicide ideation and encourage adults to understand that talking about suicide with young people is useful. Additionally, the increase of confidence levels reported following training and maintained at follow-up is likely to have been assisted by the increase of knowledge of youth mental health problems and mental health first aid strategies provided by the training.

Several limitations of this study must be noted. Firstly, the study utilised an uncontrolled design. An intervention with a randomised control group design using a larger sample size (in order to account for drop outs in the follow-up) would have been ideal for examining the impact of training on the outcome measures. However, it should be noted that the drop-out rate of participants at follow-up was dealt with using multiple imputation. In future studies, we recommend that the research protocol is communicated to the schools through embedded champions, who can then assist with reducing drop-out rates in addition to providing students with incentives to complete follow-up surveys. It is important to note that although this trial included a 3-month follow-up, a longer follow-up timeframe would potentially have given the teen and youth MHFA aiders with more opportunities to apply the knowledge and skills acquired through the training. Future research should consider a project with a 6 to 12-month follow-up arm. Finally, future evaluation studies of this nature may wish to measure the mental health status of participants as well as demographic factors such as ethnicity, length of time in Australia and years of schooling of the parents of the teens, all of which may shed further light on the social determinants of health in these groups.

Strengths of this study include being the first program of its kind that seeks to teach how to provide mental health first aid to adolescents with a CALD background. While there has been an increased emphasis on cultural competency in mental health care and the delivery of evidence-based psychosocial services for children from ethnic groups [35], to date, culturally appropriate psychoeducation initiatives at a community-based level are rare and primarily focus on adults [32, 33]. To the best of our knowledge, this adaption represents the first of its kind. The findings have demonstrated that by utilising content from the highly successful teen and Youth MHFA training curriculum and supplementing it with some culturally adapted materials, a culturally salient training program with appropriate teaching resources was developed that had significant impacts on improving MHL and help-seeking attitudes of participants.

## Conclusion

This study reports on the evaluation of the teen and Youth MHFA programs that were developed and delivered to be responsive to youth from CALD background. To the authors’ knowledge, this is the first program, aiming to equip adolescents with the skills to assist a peer who may be developing a mental health problem or experiencing a mental health crisis with a CALD focus, delivered in a culturally diverse area. Our findings indicated the training led to an improvement in a number of measures of MHL and helpful intentions of both the adolescents and adults evaluated. These results indicate that teen and Youth MHFA with a CALD focus are a recommended way of upskilling those trained and thereby leading to the improvement in youth mental health in areas with high proportion of ethnically diverse groups.

## Abbreviations

ABS: Australian Bureau of Statistics
CALD: culturally and linguistically diverse
MHFA: Mental Health First Aid
MHL: mental health literacy
NSW: New South Wales
PTSD: posttraumatic stress disorder
SWSPHN: South Western Sydney Primary Health Network
